# dbWGFP: a database and web server of human whole-genome single nucleotide variants and their functional predictions

**DOI:** 10.1093/database/baw024

**Published:** 2016-03-17

**Authors:** Jiaxin Wu, Mengmeng Wu, Lianshuo Li, Zhuo Liu, Wanwen Zeng, Rui Jiang

**Affiliations:** MOE Key Laboratory of Bioinformatics, Bioinformatics Division and Center for Synthetic & Systems Biology, TNLIST, Department of Automation, Tsinghua University, Beijing 100084, China

## Abstract

The recent advancement of the next generation sequencing technology has enabled the fast and low-cost detection of all genetic variants spreading across the entire human genome, making the application of whole-genome sequencing a tendency in the study of disease-causing genetic variants. Nevertheless, there still lacks a repository that collects predictions of functionally damaging effects of human genetic variants, though it has been well recognized that such predictions play a central role in the analysis of whole-genome sequencing data. To fill this gap, we developed a database named dbWGFP (*a database and web server of human whole-genome single nucleotide variants and their functional predictions*) that contains functional predictions and annotations of nearly 8.58 billion possible human whole-genome single nucleotide variants. Specifically, this database integrates 48 functional predictions calculated by 17 popular computational methods and 44 valuable annotations obtained from various data sources. Standalone software, user-friendly query services and free downloads of this database are available at http://bioinfo.au.tsinghua.edu.cn/dbwgfp. dbWGFP provides a valuable resource for the analysis of whole-genome sequencing, exome sequencing and SNP array data, thereby complementing existing data sources and computational resources in deciphering genetic bases of human inherited diseases.

## Introduction

The identification of genetic variants responsible for human inherited diseases is one of the major tasks in medical and human genetics (1). With the evolution of the next generation sequencing technology, it becomes more and more feasible to sequence all genetic variants in the entire human genome with low-cost in a short period of time (2,3), making whole-genome sequencing a reality in the study of human inherited diseases.

Whole-genome sequencing can typically detect much more genetic variants than the traditional SNP array technology, and many sequenced SNVs occur in low frequency or *de novo*. For example, single nucleotide variants (SNVs) detected for an individual in the 1000 genomes project is about 4 million on average, about 3–4 times more than those detected by the Affymetrix GeneChip genome-wide human SNP array 6.0. Among these SNVs, about 29% occur in low frequency (<1%). These properties, together with the fact that the number of patients and normal individuals in a whole-genome sequencing study is typically small, prohibit the direct application of such statistical genetics approaches as genome-wide association (GWA) studies (4–6) to the analysis of whole-genome sequencing data. The recent advancement in exome sequencing studies (7,8) has shown that the analysis of functionally damaging effects could be a powerful way in the identification of disease-causing SNVs (9,10). For example, we have previously demonstrated that the integration of multiple functional scores of nonsynonymous SNVs and association scores of genes hosting these SNVs by a carefully designed statistical model is effective in pinpointing pathogenic SNVs for autism, epileptic encephalopathies and intellectual disability (11,12). However, a majority of SNVs in whole-genome sequencing studies occur in non-coding regions, and there still lacks a repository that collects functional predictions and annotations of such variants. These facts have greatly restricted the scope of functional analysis of whole-genome sequencing data. Therefore, an urgent demand in whole-genome sequencing studies is to construct a database that collects functional predictions and annotations for the large number of sequenced SNVs.

There have been dozens of computational methods for predicting functionally damaging effects of nonsynonymous SNVs that occur in protein coding regions, with examples including but not limited to SIFT (13), PolyPhen-2 (14), MutationTaster (15), MSRV (16), SinBaD (17) and many others (18,19). Whole-exome predictions of these methods have also been collected in such databases as dbNSFP (20). For SNVs occurring in non-coding regions, conservation information based on multiple sequence alignment or polygenetic trees, such as GERP ++ (21), SiPhy (22), PhyloP (23), serves as a major feature for characterizing functional implications of SNVs. With the growth of functional annotations of the human genome, large-scale efforts have also been made to interpret the functional non-coding variants. For example, two leading algorithms, Combined Annotation–Dependent Depletion (CADD) (24) and Genome-Wide Annotation of VAriants (GWAVA) (25), have extended their functional predictions to non-coding variants by integrating various genomic and epigenomic annotations.

Different computational methods have their own strength and weakness, due to the reason that they use different annotations, adopt different statistical or machine learning models, and are trained with different training data. Therefore, a more comprehensive way for assessing functional implications of SNVs is to use prediction results of multiple methods to make more reliable inference. With this understanding, we developed dbWGFP, a database of whole-genome single nucleotide variants and their functional predictions. In this database, we collected nearly 8.58 billion possible human whole-genome SNVs. For each SNV, we collected 32 functional prediction scores calculated by 13 methods, 15 conservation features derived from 4 approaches, 1 sensitivity measurement and 44 valuable annotations obtained from the ENCODE project. We further compiled a cross-platform program to enable ultra-fast search of this database and offered user-friendly web services and free downloads at http://bioinfo.au.tsinghua.edu.cn/dbwgfp.

## Methods

**Figure 1.**
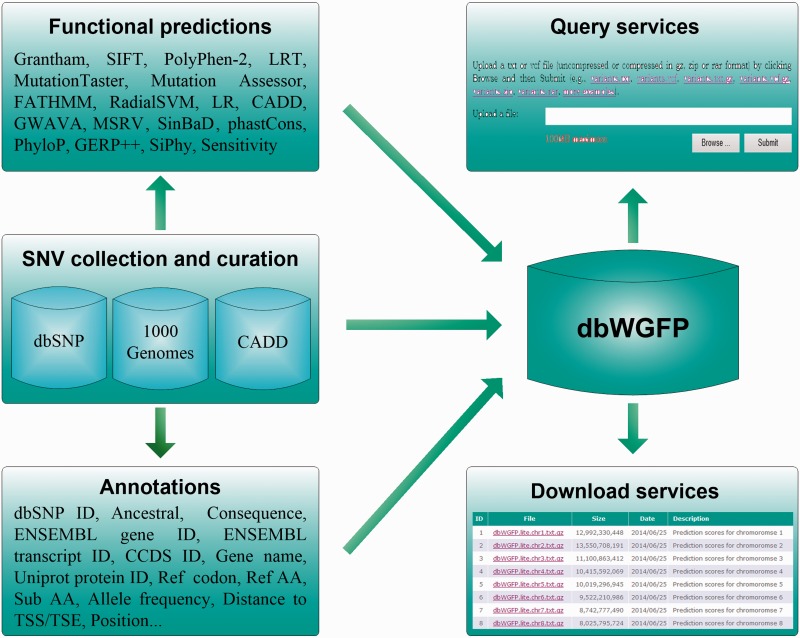
Structure of the dbWGFP database.

dbWGFP provides a well-designed database that contains 48 functional prediction scores and 44 valuable annotations for nearly 8.58 billion human SNVs. The overall structure of this database is shown in Figure 1. To meet demands of different research purposes, we offer two versions of this database. In the lite version, we only include in the database basic information of SNVs and their functional prediction scores (Table S1). In the full version, we further include annotations extracted from dbSNP (26), CADD (24), the ENCODE Project (27) and the 1000 Genomes Project (28) (Table S1). Single functional predictions have their own advantages and limitations in the scope of usage and the prediction power for different types of variants. For example, PolyPhen-2, as one of the most accurate methods for predicting functional effects of nonsynonymous SNVs, is restricted to dealing with variants located in protein coding regions, because this method calculates functional implications of SNVs based on protein sequence and structure. phastCons adopts a phylo-Hidden Markov Model (HMM) to detect conserved elements and provides a measure of conservation for nearly all possible SNVs. However, this method lacks the support of functional evidence of variants and overlooks relative importance of variants in the process of transcription and translation (21). On the other hand, current applications appeal for functional predictions of not only high accuracy but also high coverage. For example, in the widely used strategy for analyzing exome sequencing data, functional prediction scores are used to filter out variants not likely to be causative. However, exome sequencing can lead to variants in not only protein coding regions but also such flanking regions as promoters and splice sites. With this understanding, we try to construct a database that includes as many functional prediction scores and annotations as possible. Specifically, we collect functional prediction scores that meet two standards. First, the method for calculating a prediction score should be formally published. Second, the method should provide a website for downloading pre-calculated scores or a software package for calculating scores. With these criteria, we collected 48 functional prediction scores that were derived from 17 methods. Among them, scores of MSRV and SinBaD were calculated by using their software, and the other scores were downloaded from websites.

### Collection and curation of SNVs

We collected all possible SNVs in the human genome by integrating those occurring at least once in dbSNP (26), dbNSFP (20,29) and CADD (24). By doing this, we obtained a total of 8 576 251 873 human SNVs based on the GRCh37/hg19 reference. We then extracted annotations for these SNVs from dbSNP, CADD, the ENCODE Project and the 1000 Genomes Project, including consequence type, corresponding codons and genes, allele frequencies, positions, distance to splicing site and many other properties. 

### Extraction of functional prediction scores

We collected 48 functional prediction scores for each SNV, including 32 functional features, 15 conservation features and 1 sensitivity measurement. The 32 functional features are calculated by 13 popular functional prediction methods, including Grantham (30), SIFT (13,31), PolyPhen-2 (14), LRT (18), MutationTaster (15), Mutation Assessor (19), FATHMM (32), RadialSVM (29), LR (29), CADD (24), GWAVA (25), MSRV (16) and SinBaD (17). The 15 conservation features are derived by 4 conservation calculation approaches, including phastCons (33), PhyloP (23), GERP ++ (21) and SiPhy (22). The only sensitivity measurement describes subgroups of non-coding categories that share almost the same selective constraint as coding genes(34). Details about these functional prediction scores are summarized in Table 1 and described briefly as follows.

**Table 1.** Computational methods for predicting functionally damaging effects or conservation properties of single nucleotide variants

Method	Version	Source	Website
Grantham	Sep-74	CADD	—
SIFT	Aug-11	dbNSFP	http://sift.jcvi.org
PolyPhen-2	v2.2.2	dbNSFP	http://genetics.bwh.harvard.edu/pph2
LRT	Nov-09	dbNSFP	http://www.genetics.wustl.edu/jflab/lrt_query.html
MutationTaster	Mar-13	dbNSFP	http://www.mutationtaster.org
Mutation Assessor	Release 2	dbNSFP	http://mutationassessor.org
FATHMM	v2.3	dbNSFP	http://fathmm.biocompute.org.uk
RadialSVM	v2.4	dbNSFP	http://sites.google.com/site/jpopgen/dbNSFP
LR	v2.4	dbNSFP	http://sites.google.com/site/jpopgen/dbNSFP
CADD	v1.0	CADD	http://cadd.gs.washington.edu
GWAVA	v1.0	GWAVA	https://www.sanger.ac.uk/sanger/StatGen_Gwava
MSRV	Aug-07	MSRV	http://bioinfo.au.tsinghua.edu.cn/msrv
SinBaD	Nov-12	SinBaD	http://tingchenlab.cmb.usc.edu/sinbad
phastCons	Nov-09	UCSC	http://hgdownload.soe.ucsc.edu/goldenPath/hg19/phastCons46way
PhyloP	Nov-09	UCSC	http://hgdownload.soe.ucsc.edu/goldenPath/hg19/phyloP46way
GERP ++	May-11	GERP	http://mendel.stanford.edu/SidowLab/downloads/gerp
SiPhy	v0.5	SiPhy	http://www.broadinstitute.org/genome_bio/siphy

We extracted SIFT, PolyPhen-2, LRT, MutationTaster, Mutation Assessor, FATHMM, RadialSVM and LR scores from the dbNSFP database (Version 2.4). These scores measure functional changes of the encoded protein for a nonsynonymous SNV, whose occurrence may results in the change of amino acid and potentially affects protein structure and function. Briefly, SIFT takes advantage of the position-speciﬁc probability estimation using PSSM with Dirichlet priors to estimate whether the altered amino acid affects protein function (13). The smaller the SIFT score, the more likely the SNV could destroy the function of the protein. PolyPhen-2 calculates a set of features for a SNV based on the encoded protein sequence and protein structure, and trains a naïve Bayes model coupled with entropy-based discretization to identify the structural and functional effect of the SNV (14). Based on the null hypothesis that each codon is evolving neutrally with no difference in the rate of nonsynonymous to synonymous substitution, LRT adopts the log likelihood ratio of the conserved relative to neutral model to predict the deleteriousness of a SNV (18). Similar to SIFT, the smaller the LRT score, the more likely a SNV would destroy the function of the protein. MutationTaster computes a large number of sequence-based features and trains a naïve Bayes classifier to predict the potential deleterious nonsynonymous SNVs (15). Due to evolutionary conservation of the affected amino acid in protein homolog, Mutation Assessor evaluates the functional effect of the SNV resulting in the amino acid change (19). Mutation Assessor can predict both somatic mutations discovered in cancers or missense SNVs. FATHMM relies on a hidden Markov models to predict the functional, molecular and phenotypic effect of missense variants or cancer-associated variants (32). RadialSVM and LR are merged prediction scores that are derived by using SVM and logistic regression respectively to integrate 10 existing prediction scores (SIFT, PolyPhen-2 HDIV, PolyPhen-2 HVAR, GERP ++, MutationTaster, Mutation Assessor, FATHMM, LRT, SiPhy, PhyloP) and the maximum allele frequency in the 1000 Genomes Project (29).

We downloaded the Grantham and CADD scores from the CADD website (Version 1.0). The Grantham score indicates differences of physicochemical properties between amino acids, and the larger the difference score, the more likely a SNV would destroy the function of the host protein (30). The CADD score is obtained by integrating annotations from Ensembl Variant Effect Predictor (VEP) (35), ENCODE Project (27) and UCSC Genome Browser tracks (36) to prioritize whole-genome functional variants. CADD provides two types of prediction scores: the raw score with high resolution and the scaled score that is easier to interpret and comparable across different CADD versions or models.

We downloaded GWAVA score from its website (25). This method predicts the functional effect of non-coding genetic SNVs based on sequence-based properties and a large set of annotations of non-coding elements from the ENCODE and GENCODE projects (37). We downloaded PhastCons and PhyloP scores from the UCSC Genome Browser. PhastCons uses a hidden Markov model to predict the probability that a SNV belongs to a conserved element based on the multiple sequence alignment of the human genome against other species (33). PhyloP computes an exact *p*-value under a continuous Markov substitution model to estimate the interspecies conservation for each SNV (23). We downloaded SiPhy scores from the public ftp site of the Board Institute. SiPhy takes advantage of rigorous statistical tests to identify bases under selection constraint based on multiple sequence alignment with 29 mammals. SiPhy also estimates stationary distribution of different nucleotides at a site (22). GERP adopts maximum likelihood evolutionary rate estimation to calculate position-specific estimates of evolutionary constraint (38). GERP ++, an advanced version of GERP, uses a more rigorous set of algorithms to calculate position-specific ‘rejected substitutions’ scores and to indentify evolutionarily constrained elements (21). GERP ++ neutral evolution scores, rejected substitution scores, element scores and element *p*-values were all downloaded from the GERP website.

We calculated MSRV and SinBaD scores by using software packages provided by these methods. Briefly, MSRV applies an ensemble learning approach with a set of 24 physiochemical properties and 2 conservation scores to prioritize disease-causing nonsynonymous SNVs (16). SinBaD adopts a logistic regression model with 90 binary features obtained from multiple sequence alignment (17) to quantitatively measure functional effects of mutations in not only protein coding regions but also promoter regions and introns.

### Extraction of annotations

In the full version of dbWGFP, we further collected 44 useful information or annotations from dbSNP, CADD, the ENCODE Project and the 1000 Genomes Project. First, we included basic information for each SNV and its corresponding codons and genes, including reference SNP ID, ancestral base, annotation type, consequence type of the variants, ENSEMBL gene ID, ENSEMBL transcript ID, CCDS ID, gene name, protein accession number and ID in the UniprotKB database (39), reference codon, reference and substituted amino acids. Second, we extracted from the 1000 Genomes Project related annotations, including the validated status, project phase, common variant or not, and different types of allele frequency for different type of populations. Finally, we included from CADD or the ENCODE Project such annotations as distance to the closest Transcribed Sequence Start (TSS), distance to the closest Transcribed Sequence End (TSE), amino acid position, codon position, base position from transcription start, relative position in transcript, base position from coding start, relative position in coding sequence, distance to splice site, closest splice site is ACCEPTOR or DONOR, total number of exons, and total number of introns.

### Coverage and correlation of functional scores

Different types of functional scores are designed for different types of variants. For example, SIFT and PolyPhen-2 can only predict the deleteriousness of nonsynonymous SNVs, while CADD and GERP ++ can give estimations of functional effects for SNVs across the whole genome. Therefore, we summarized the coverage of each functional effect score for each chromosome in Table 2. From the table, we can see that CADD and the four conservation scores have high coverage, while functional prediction scores designed only for nonsynonymous SNVs, including SIFT, PolyPhen-2, LRT, MutationTaster, Mutation Assessor, FATHMM, RadialSVM, LR, MSRV and SinBaD, have low coverage.

**Table 2.** Coverage of functional prediction scores in percentage

Chromosome	1	2	3	4	5	6	7	8	9	10	11	12	13	14	15	16	17	18	19	20	21	22	X	Y	All
Grantham	1.15	0.79	0.76	0.55	0.66	0.78	0.77	0.6	0.86	0.77	1.15	1.04	0.48	0.96	1.1	1.4	1.88	0.54	2.97	1.02	0.72	1.59	0.64	0.24	0.9
SIFT	1.16	0.8	0.75	0.55	0.66	0.78	0.76	0.61	0.87	0.78	1.13	1	0.46	0.91	1.11	1.37	1.89	0.54	3.05	1.04	0.71	1.57	0.62	0	0.89
PolyPhen-2	1.09	0.74	0.73	0.51	0.63	0.74	0.71	0.57	0.82	0.73	1.08	0.98	0.46	0.86	1.01	1.28	1.76	0.51	2.84	0.98	0.68	1.47	0.59	0.02	0.84
LRT	1.03	0.67	0.7	0.51	0.59	0.72	0.66	0.55	0.78	0.72	1.02	0.94	0.46	0.83	0.97	1.2	1.66	0.47	2.09	0.97	0.63	1.39	0.56	0.01	0.79
MutationTaster	1.26	0.87	0.84	0.6	0.72	0.85	0.83	0.67	0.95	0.85	1.25	1.14	0.52	1.01	1.15	1.45	2.01	0.58	3.12	1.13	0.8	1.7	0.7	0.02	0.97
Mutation Assessor	1.06	0.73	0.7	0.51	0.62	0.72	0.69	0.54	0.8	0.7	1.05	0.95	0.45	0.84	1	1.22	1.72	0.51	2.74	0.95	0.65	1.41	0.57	0.03	0.82
FATHMM	1.03	0.71	0.68	0.49	0.6	0.7	0.67	0.52	0.77	0.68	1.02	0.93	0.43	0.8	0.98	1.18	1.64	0.49	2.64	0.92	0.64	1.34	0.57	0.03	0.79
RadialSVM	1.18	0.81	0.77	0.56	0.67	0.79	0.78	0.62	0.88	0.79	1.17	1.05	0.48	0.94	1.08	1.37	1.88	0.54	3.01	1.05	0.74	1.6	0.65	0.05	0.91
LR	1.18	0.81	0.77	0.56	0.67	0.79	0.78	0.62	0.88	0.79	1.17	1.05	0.48	0.94	1.08	1.37	1.88	0.54	3.01	1.05	0.74	1.6	0.65	0.05	0.91
CADD	100	100	100	100	100	100	100	100	100	100	100	100	100	100	100	100	100	100	100	100	100	100	100	100	100
GWAVA	0.52	0.53	0.54	0.55	0.53	0.55	0.55	0.58	0.54	0.55	0.56	0.54	0.54	0.54	0.53	0.62	0.53	0.55	0.6	0.59	0.59	0.58	0.36	0.11	0.53
MSRV	0.99	0.66	0.67	0.47	0.6	0.68	0.66	0.51	0.76	0.68	0.99	0.91	0.42	0.79	0.94	1.17	1.66	0.48	2.56	0.92	0.65	1.33	0.56	0.01	0.78
SinBaD	1.28	0.88	0.84	0.61	0.73	0.86	0.84	0.68	0.96	0.86	1.26	1.15	0.53	1.02	1.18	1.49	2.04	0.59	3.24	1.14	0.81	1.72	0.7	0.05	0.99
phastCons	99	99	99	99	98	99	98	98	98	99	98	98	99	99	99	98	99	99	97	99	99	98	95	85	98
PhyloP	99	99	99	99	98	99	98	98	98	99	98	98	99	99	99	98	99	99	97	99	99	98	95	85	98
GERP ++	100	100	100	100	100	100	100	100	100	100	100	100	100	100	100	100	100	100	100	100	100	100	100	100	100
SiPhy	98	98	98	98	98	98	97	98	98	98	97	98	99	98	98	97	98	98	95	98	96	97	92	0	97

**Figure 2.**
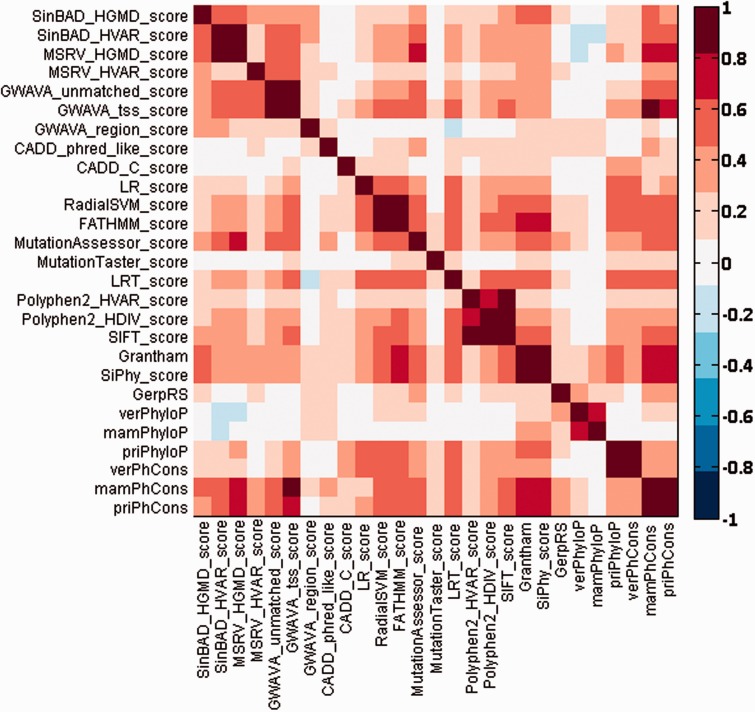
Pairwise Spearman’s rank correlation coefficients between different functional prediction scores.

Different types of prediction methods give different functional effect scores for the same SNV. Therefore, we checked pairwise agreement between different prediction scores for SNVs occurring in chromosome 22 by using the Spearman’s rank correlation coefficient, and we summarized the results in Figure 2. From this figure, we can see that most prediction scores have medium to high correlations with a few other scores. For example, prediction scores of MSRV are highly correlated with those of SinBaD, and scores of PhastCons are highly correlated with those of GWAVA. Nevertheless, there also exist some scores (e.g. MutationTaster) that have low correlations with the others.

### Comparison of the prediction power between different scores

dbWGFP contains 15 conservation features derived by 4 conservation calculation approaches and 32 functional features calculated by 13 popular functional prediction methods. Seven of these methods (phastCons, PhyloP, GERP ++, SiPhy, Grantham, CADD and GWAVA) intend to provide prediction scores for variants spreading over the whole genome. The other ten methods (SIFT, PolyPhen-2, LRT, MutationTaster, Mutation Assessor, FATHMM, RadialSVM, LR, MSRV and SinBaD) only focus on variants in protein coding regions. In order to obtain a comprehensive understanding about the prediction power of these methods, we collected a set of disease-causing SNVs from the HGMD database and a set of neutral SNVs from the 1000 Genomes Project. The disease-causing variants, used as positive cases, were further partitioned into 52 007 protein coding SNVs, 8822 splicing SNVs and 1811 regulatory SNVs. Accordingly, the neutral variants, used as negative controls, were also partitioned into 272 534 protein coding SNVs, 2897 splicing SNVs, and 701 984 regulatory SNVs. For each of these variants, we extracted the conservation scores and functional scores from the dbWGFP database, obtaining a total of 17 scores.

Focusing on scores that cover at least 5% of SNVs in a category. We first performed a t-test to see whether a prediction score is significantly different between positive and negative SNVs. Results, as shown in Table 3, suggest that all the 17 scores are significantly different between the two class of SNVs in protein coding regions. For SNVs in splice sites, only 8 scores cover 5% or more SNVs. Within these scores, SinBaD has the highest power in discriminating disease causing variants against neutral ones. For SNVs in regulatory regions, only 5 scores cover 5% or more SNV, and GWAVA has the highest discriminant power.

**Table 3.** Prediction power of the scores

Type of SNVs	Coding SNVs			Splicing SNVs			Regulatory SNVs		
#(disease SNVs)	52007			8822			1811		
#(neutral SNVs)	272534			2897			701984		

Method	*p*-Value	AUC	Coverage(%)	*p*-Value	AUC	Coverage(%)	*p*-Value	AUC	Coverage(%)

mamPhCons	0	67.22	99.99	5.99E-21	56.89	99.99	1.18E-57	56.89	99.78
mamPhyloP	0	64.59	100	0.0264	51.61	99.99	1.12E-36	58.6	99.8
GERP ++	0	66.43	100	3.27E-08	56.36	100	4.78E-19	58.73	100
SiPhy	0	56.23	99.94	1.8E-17	55.54	99.91	1.91E-45	60.13	99.23
Grantham	0	60.37	96	–	–	–	–	–	–
CADD	0	77.31	100	2.44E-21	55.64	100	2.97E-91	65	100
GWAVA	5.74E-66	54.05	88.93	0.0005	53.37	36.35	7.73E-277	81.61	99.67
SIFT	4.33E-308	64.38	93.55	–	–	–	–	–	–
Polyphen2	0	77.04	93.36	–	–	–	–	–	–
LRT	0	70.94	86.09	–	–	–	–	–	–
MutationTaster	0	63.46	99.07	1.15E-08	53.07	72.33	–	–	–
MutationAssessor	0	77.55	94.12	–	–	–	–	–	–
FATHMM	0	86.41	90.27	–	–	–	–	–	–
RadialSVM	0	87.79	96.28	–	–	–	–	–	–
LR	0	87.96	96.28	–	–	–	–	–	–
MSRV	0	80.56	89.81	–	–	–	–	–	–
SinBAD	0	74.16	99.38	1.06E-195	70.48	72.58	–	–	–

We then explored the ability of each score in predicting disease-causing SNVs. For this purpose, we varied the decision threshold for a score and calculated the sensitivity and specificity at each threshold value. Here, the sensitivity is defined as the fraction of positive SNVs whose scores exceed a threshold, and the specificity is defined as the fraction of negative SNVs whose scores do not exceed a threshold. We then plotted the receiver operating characteristic curve (sensitivity versus 1-specificity) and calculated the area under this curve (AUC). Results, as shown in Table 3, suggest that the performance of different methods is quite different. For SNVs in protein coding regions, LR has the highest performance, followed by RadialSVM, FATHMM, MSRV and CADD, respectively. For SNVs in splice sites, SinBAD outperforms all the other methods. For SNVs in regulatory sites, GWAVA has the highest performance. This comprehensive comparison of the prediction power between different scores therefore provides insightful understanding in the determination of suitable prediction scores in real applications. Overall, the prediction of disease-causing SNVs in splice sites and regulatory regions are much harder than that in protein coding regions, because the AUCs of the former two categories are typically much lower than those of the later class. Such an observation suggests the urgent demand of developing an effective computational tool for predicting functionally damaging effects of variant in non-coding regions.

The coverage of different types of prediction scores varies significantly, resulting in the missing data problem. To address this problem, we propose the following three methods. First, users can completely ignore missing data and only focus on scores of complete information. Second, users can adopt a statistical or machine learning approach that can easily handle missing data. Fisher’s method and Naïve Bayes are two examples. Third, users can adopt a strategy to impute missing values and then use the data as if they were observed completely. In addition, although some prediction scores are highly correlated, there do exist scores of low correlations with the others, leading to potential conflict between the scores. To account for this issue, we propose the following three strategies. First, as a stringent way, users can define a SNV as functional only if all the prediction scores indicate the functionally damaging effect of the SNV. Second, as a loose option, users can define a SNV as functional if any of the prediction scores indicate its functionally damaging effect. Certainly, these two strategies are either too rigorous or too loose. Therefore, a more reasonable way is to comprehensively consider all the prediction results and determine the functionally damaging effect of a SNV by using the majority voting rule.

## Software

dbWGFP offers a user-friendly web interface to facilitate the access of the database. The web interface provides two main components: a query service for retrieving functional prediction scores and annotations of SNVs in different data formats and a download service for setting up a local version of this database. In the step-by-step mode of the query service, users can upload a file containing query variants and retrieve results online. In the batch query mode, users can upload a file containing query variants and an email address. A URL of the query results will then be send via email. dbWGFP provides two versions for downloading. The lite version includes prediction scores of human whole-genome SNVs. The full version includes both prediction scores and annotations. Both versions include a search program that can retrieve predictions and/or annotations in a highly efficient way. Different versions of dbWGFP are also archived for easy access.

### Ultra-fast search program

Sequentially scanning dbWGFP to retrieve a query SNV is prohibited due to the huge number of SNVs collected in this database. Therefore, we developed a highly efficient search program to enable ultra-fast locating of a SNV in the database. In order to test the speed of the search program, we selected an individual (HG00096) at random from the 1000 Genomes Project, extracted a total of 3 844 226 SNVs occurring in the whole genome of this individual, and applied the search program to retrieve predictions and annotations from dbWGFP. The results are summarized in Table 4. From the table, we can see that for the lite version of dbWGFP, our search program, when using 8 threads simultaneously, can efficiently deal with queries at the speed of 4999 SNVs per second, and it takes only 769 s to obtain functional predictions for SNVs spreading across the whole genome of a human. For the full version of dbWGFP, our search program can efficiently deal with queries at the speed of 3647 SNVs per second, and it takes only 1054 s to obtain both functional predictions and annotations for SNVs spreading across the whole genome of a human. We also notice that the running time for taking all variants as a single query file is significantly shorter than the summation of running time for taking individual chromosomes as separate query files. This phenomenon is due to the fact that multiple threads read separate database files for different chromosomes in the former case. Hence, we suggest users combining their data for different chromosomes into a single query file to maximize the search performance (Table 4).

**Table 4.** Running time of the dbWGFP search program. Results are obtained using 8 threads in a server with dual Intel E5-2630V2 CPU (2.6 GHz) and 64GB memory

**Chromosome**	**#(SNVs)**	**Lite version**		**Full version**	
		**Time (s)**	**#(SNPs)/second**	**Time (s)**	**#(SNPs)/second**
1	291 183	86	3386	116	2510
2	306 260	89	3441	121	2531
3	265 905	76	3499	103	2582
4	281 093	72	3904	96	2928
5	240 036	86	2791	94	2554
6	254 105	67	3793	89	2855
7	214 802	58	3703	83	2588
8	201 101	58	3467	73	2755
9	159 777	48	3329	68	2350
10	192 012	54	3556	74	2595
11	194 987	54	3611	75	2600
12	176 087	53	3322	76	2317
13	147 631	42	3515	58	2545
14	124 626	37	3368	52	2397
15	110 700	36	3075	48	2306
16	114 626	35	3275	52	2204
17	102 123	36	2837	47	2173
18	111 964	36	3110	46	2434
19	84 735	26	3259	38	2230
20	77 334	28	2762	37	2090
21	55 667	17	3275	23	2420
22	48 737	16	3046	25	1949
X	88 735	46	1929	57	1557
Combined	3 844 227	769	4999	1054	3647

### Query service

**Figure 3.**
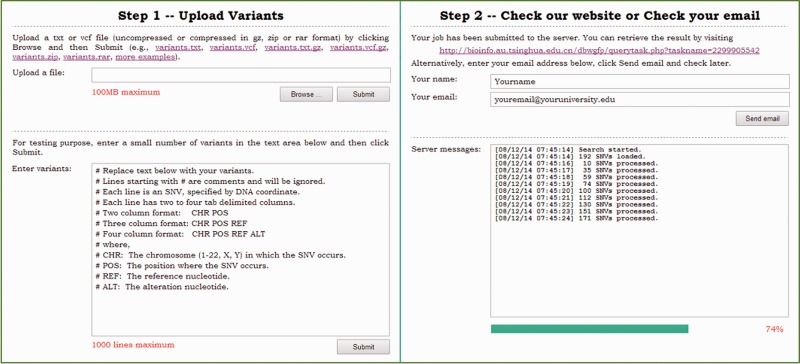
Illustration of the step-by-step mode of the query service.

The query service provides two accessing modes. In the step-by-step mode illustrated in Figure 3, a user can upload a file containing query SNVs and then check the web site for results. In the batch mode, a user can upload an archive including query SNVs and an email address, and then check email for results later. In either mode, a query file typically includes multiple lines, each of which is given in one of the following four formats. First, a query line can be given as two column text (‘chr pos’). In this case, the server locates the query position in the query chromosome and output predictions and annotations of all possible SNVs in the query position. Second, a query line can be given as three column text (‘chr pos ref’). In this case, the server locates the query position in the query chromosome and output predictions and annotations of all possible SNVs that occur in the query position and whose reference nucleotide is identical to the query. Third, a query line can be given as four column text (‘chr pos ref alt’). In this case, the server outputs predictions and annotations of the SNV defined by the query. Finally, a query line can be given in vcf format. In this case, the server also outputs predictions and annotations of the SNV defined by the query. Considering that in a real whole-genome sequencing study, the number of SNVs is typically huge, the dbWGFP web service also accepts input files compressed in gz, bz2, zip or rar formats. Similarly, output files are also given in these compressed formats.

### Download service

The download service allows a user to download parts or the entire dbWGFP database. For both the lite and the full versions, we partitioned SNVs according to chromosomes and provided files compressed in gz format for individual chromosomes. We further generated a single compressed archive file for each version.

## Conclusions and discussion

In this paper, we have introduced dbWGFP, a database and web server of human whole-genome single nucleotide variants and their functional predictions. This database collects nearly 8.58 billion possible SNVs across the whole human genome, with each SNV described by 48 functional prediction scores and 44 valuable annotations. To the best of our knowledge, dbWGFP is the first large-scale comprehensive database for functional predictions and annotations of human whole-genome SNVs.

This database can not only be helpful in the capture of causative variants from massive candidates derived from whole-genome or exome sequencing data, but also provide a valuable resource in the study of human genetic variants. For example, after sequencing the whole genome of one or a few patients, a bunch of candidate SNVs can be extracted from the sequencing data. Given all the candidate SNVs as input, dbWGFP can be used to effectively collect functional prediction scores and annotations for each candidate SNV. Based on these scores and annotations, researchers could filter out a large set of neutral SNVs that are believed to have little functional effect, and obtain the remaining functional SNVs for further study. Similarly, dbWGFP can also be used in the analysis of exome sequencing or SNP array data, thereby complementing existing data sources and statistical methods in deciphering genetic bases of human inherited diseases.dbWGFP can be further improved from the following aspects. First, currently computational methods for predicting functional effects of whole-genome variants are still quite limited, since scientists just begin to make such efforts recently. As more prediction approaches become available in the near future, more available functional prediction scores can be incorporated into our database. Second, important gene annotations and protein annotations can also be included in our database. These annotations may include but not limited to gene annotations from Gene Ontology (40), protein-protein interaction network from STRING (41), pathway information from KEGG (42) and many others. Third, phenotypic properties for human whole-genome SNVs can also be included in our database. These properties can be extracted from existing databases such as OMIM (43), HGMD (44) and COSMIC (45). The inclusion of such phenotypic information may further improve the inference of causative variants for human inherited diseases, as we have done in our previous studies for prioritizing candidate genes (46). Finally, although we focus on single nucleotide variants in the current release of dbWGFP, it is obvious that other types of variants such as small insertion or deletion can also be included in the future.

## Supplementary Material

Supplementary Data
