# Supplementary material for: dbWGFP: a database and web server of human whole-genome single nucleotide variants and their functional predictions
Source: Database (Oxford). 2016 Mar 17;2016:baw024. doi: 10.1093/database/baw024 (PMC4795934; doi:10.1093/database/baw024)
Supplement: Supplementary Data [file supp_2016_baw024_index.html]

Supplementary Data 

# dbWGFP: a database and web server of human whole-genome single nucleotide variants and their functional predictions

## Supplementary Data

files

- Supplementary Data - xls file
